# Harry Kaye Rose (LRCP, LRCS, DPH, DPM, FRCPsych): Formerly Consultant Psychiatrist, Greenwich District Hospital and Bexley Hospital

**DOI:** 10.1192/pb.bp.115.051359

**Published:** 2016-02

**Authors:** The Rose Family, Brian Humblestone, Tony Mansi

**Figure:**
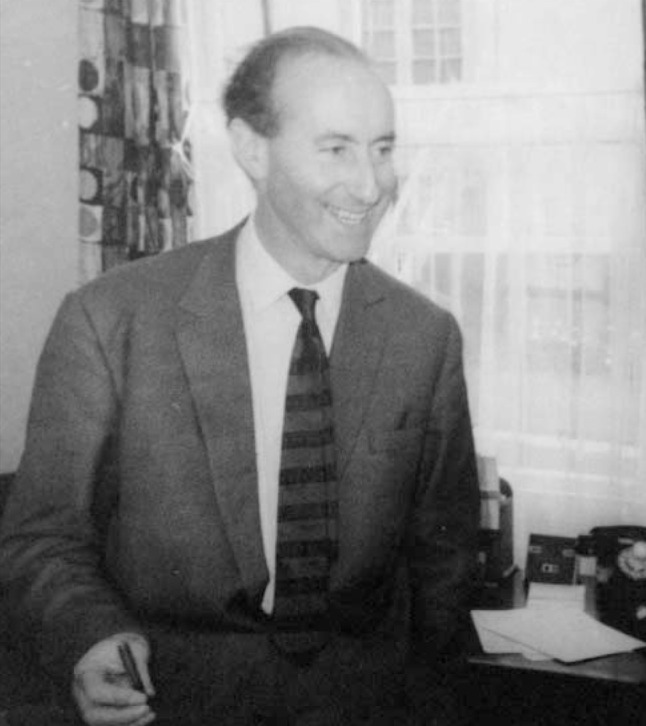


A spell of service in the Merchant Navy provided Harry Kaye Rose, who has died aged 89 years, with the eye-opener that led him to develop an interest in alcoholism and a realisation of its importance to psychiatry. He worked closely with Dr Max Glatt in the 1950s when the study of the subject was in its infancy. He was instrumental in setting up one of the few centres of excellence in Warlingham Park Hospital. The treatment model that he and Max Glatt developed was to be the forerunner of that employed in today's Priory clinics. They also established the first treatment centres for drug and alcohol misuse in the prison service. In the early 1960s, drawing on his own experiences at sea, Dr Rose published with Dr Glatt influential academic papers on the health of the merchant seaman and alcoholism.

After leaving Warlingham Park Hospital, he was appointed to be the senior consultant in psychiatry at the new Greenwich District Hospital. He undertook regular consultancy work at the nearby Mabledon and Greenwich hospitals, and was a consultant in forensic psychiatry for the Home Office for several years.

At the Mabledon Hospital he worked with George Bram, treating traumatised and shell-shocked Polish refugees and soldiers who could not return to Poland after the Second World War. It was here that he struck up a friendship with one of Field Marshall Józef Piłsudski's daughters, Wanda.

In addition to his work in the National Health Service, Dr Rose had an extensive private practice with a patient load that read like a selection from *Who's Who*. He treated government ministers, members of the House of Lords, television and film stars, chief executives of FTSE 100 companies, rock stars and royalty as well as some of the most dangerous and notorious criminals in the country. He also did a great deal of work pro bono for people who were unable to afford treatment.

Dr Rose came from a middle class Jewish family in Glasgow and won many undergraduate prizes in medicine at the University of Glasgow Medical School before qualifying in 1947. He was an outstanding student who gained many awards and prizes during his time at university, including two gold medals. He did his national service as a Captain in the Royal Army Medical Corps, mostly in Colchester. Desperate to travel, he was disappointed that he failed to obtain an overseas posting and, on discharge from the army, joined the Merchant Navy as a ship's surgeon. He travelled the world on both mixed-passenger and cargo vessels. One of his duties was to entertain the passengers, so he had his own table, having to sit through two sittings of seven-course meals every day. It was here that he learnt his extraordinary powers of dietary self-control, later to stand him in good stead when he developed diabetes in his fifties.

After retirement he settled in Devon where he lived for his last 9 years. He died on 30 September 2013, leaving his wife, a daughter, a son, and three grandchildren.

